# Dapagliflozin improves podocytes injury in diabetic nephropathy via regulating cholesterol balance through KLF5 targeting the ABCA1 signalling pathway

**DOI:** 10.1186/s13098-024-01271-6

**Published:** 2024-02-07

**Authors:** Jingshu Sun, Xinyu Zhang, Simeng Wang, Dandan Chen, Jianqiang Shu, Nannan Chong, Qinglian Wang, Ying Xu

**Affiliations:** 1 Shandong Provincial Hospital, Shandong University, Jinan, Shandong China; 2 Department of Nephrology, Weifang People’s Hospital, Weifang, Shandong China; 3 Department of Nephrology, Shandong Provincial Hospital Affiliated to Shandong First Medical University, Jinan, Shandong China

**Keywords:** Dapagliflozin, Diabetic Nephropathy, Cholesterol, KLF-5, ABCA1

## Abstract

Diabetic nephropathy (DN), one of the more prevalent microvascular complications in patients diagnosed with diabetes mellitus, is attributed as the main cause of end-stage renal disease (ESRD). Lipotoxicity in podocytes caused by hyperglycemia has been recognised as a significant pathology change, resulting in the deterioration of the glomerular filtration barrier. Research has demonstrated how dapagliflozin, a kind of SGLT2i, exhibits a multifaceted and powerful protective effect in DN, entirely independent of the hypoglycemic effect, with the specific mechanism verified. In this present study, we found that dapagliflozin has the potential to alleviate apoptosis and restore cytoskeleton triggered by high glucose (HG) in vivo and in vitro. We also discovered that dapagliflozin could mitigate podocyte cholesterol accumulation by restoring the expression of ABCA1, which is the key pathway for cholesterol outflows. This research also mechanistically demonstrates that the protective effect of dapagliflozin can be mediated by KLF-5, which is the upstream transcription factor of ABCA1. Taken together, our data suggest that dapagliflozin offers significant potential in alleviating podocyte injury and cholesterol accumulation triggered by high glucose. In terms of the mechanism, we herein reveal that dapagliflozin could accelerate cholesterol efflux by restoring the expression of ABCA1, which is directly regulated by KLF-5.

## Introduction

Diabetic nephropathy (DN) remains the most common cause of end-stage renal disease (ESRD), accounting for about half of all diagnoses [1]. Despite the significant advances in our understanding of the underlying pathophysiologic mechanisms of DN over the past few decades, many details remain unknown regarding the development and progression of diabetic renal injury [2]. Podocytes are epithelial cells of the glomerular, which are also a primary target in the development of DN. The first symptom of DN is microalbuminuria, which is often attributed to podocyte injury. Studies among DN population and animal models have also revealed that the progressive glomerulopathy in DN is closely related to the integrity of the glomerular filtration barrier (GFB) and the loss of podocytes [3, 4]. As terminally-differentiated cells, the loss of podocytes often resulted from apoptosis and previous studies have identified increased podocyte apoptosis as an important factor in the pathogenesis of DN.

A growing body of evidence continued to demonstrate that renal lipid accumulation, especially cholesterol accumulation, is likely to contribute to kidney dysfunction [5–7]. Accordingly, the cholesterol balance in podocytes has been the focus of research for many years. Yet despite this, an accurate understanding of podocytes’ cholesterol metabolism under diverse physiological and pathophysiological conditions remains ambiguous [8]. Cholesterol homeostasis is essential for maintaining cellular function. These unique terminally-differentiated cells reside in the urinary space with their elaborate cytoarchitecture-slit diaphragm formed by the foot process. These slit diaphragm formations of podocytes represent a lipid-raft-like structure in which essential podocyte proteins, such as transmembrane proteins (nephrin), integral membrane proteins (podocin), structural proteins (α-actinin-4), signalling adaptors (CD2AP), and ion channels (TRPC6) are organised into a multiprotein complex. In some cases, the nature of their interaction is predicated upon cholesterol (podocin dependent activation of TRPC6), suggesting that lipids, especially cholesterols, might play a decisive role in assuring proper localisation and function of slit diaphragm proteins, which could be perturbed in cellular lipid/cholesterol imbalances, among other pathomechanistic sequels. Cholesterol homeostasis comprises de novo cholesterol synthesis at the endoplasmic cellular and efflux of cholesterol. Research has revealed that cholesterol efflux is vital for podocytes in the DN model, which is controlled by ABCA1/ABCG1. Promoting cholesterol efflux by upregulated ABCA1/ABCG1 would thus improve podocyte function.

Dapagliflozin, a kind of SGLT2 inhibitor, is a new classical agent that has shown potential for reducing the reabsorption of glucose in renal proximal tubules [9]. Recently, research has focused on ascertaining the protective effect of SGLT2i on diabetic nephropathy and non-diabetic chronic kidney diseases, with positive results being found in large RCTs. In terms of the podocyte protective effect, it is independent of its hypoglycemic effect in diabetic nephropathy. Nonetheless, the detailed protective mechanisms of dapagliflozin on podocytes remain unclear. In our study, we aim to verify the effect of dapagliflozin on podocyte injury and further explore the specific mechanisms as to whether its protective effect is based on reducing lipid accumulation.

## Materials and methods

### Chemicals and reagents

Dapagliflozin was purchased from the MedChemExpress Bio-Technology company (HY-10,450). A 10 mM stocking solution was dissolved and stored at -80˚C. RPMI 1640 medium and foetal bovine serum (FBS) were purchased from Gibco, Invitrogen (Carlsbad, CA, USA). Trypsin/EDTA was purchased from HyClone (Logan, UT, USA). WST-8 cell proliferation and cytotoxicity assay kit was purchased from Dojindo (CK04, Kumamoto, Japan). A cholesterol detection assay kit was purchased from Beijing Applygen Technologies (E-1015). The phalloidine staining reagent was purchased from the Sigma-Aldrich company. Nile acid was purchased from Macklin Inc (49,409). The siRNA specific for KLF-5 was designed and provided by the Genomeditech Company (Shanghai). Reagents for real-time PCR were purchased from Takara.

Primary antibodies are detailed as follows: Anti-ABCA1 (Abcam, Cambridge, MA, UK) for WB and IHC. Anti-KLF 5 (Abclonal, China) for WB and IHC. Anti-podocin (Abcam, UK); anti-nephrin (Abcam, UK); anti-LDLR (Abcam, UK); Anti-HMGCR (Abcam, UK); anti-podocin (Abcam, UK); anti-Bax (Abcam, UK); anti-Bcl-2 (Abcam, UK); anti-caspase 3 (Abcam, UK); and, anti-GAPDH (Proteintech, China). Rats were purchased from the Animal Experimental Centre of Shandong University. All other materials and reagents were endotoxin-free and supplied by the central lab of Shandong Provincial Hospital.

### Cell culture

Human podocytes were obtained from Professor Fan Yi (Shandong University) and cultured in RPIM 1640 medium with 10% FBS. The medium was supplemented with 10 U/ml interferonγ and enclosed within a humidified incubator with 5% CO2 at 33˚C, so as to induce proliferation. Once prepared for treatment, cells were transferred to an incubator with 5% CO2 at 37˚C without interferonγ, in order to induce differentiation, where it stayed for at least 14 days. In the following experiments, different mediums were designed and incubated with cells that were low glucose (LG, 5.5 mM glucose), high glucose (HG, 30 mM glucose), and high glucose plus variable concentrations of dapagliflozin. Prior to stimulation, cells were arrested in a medium with 1% FBS for 12 h. The quiescent cells were then treated with high glucose or high glucose plus dapagliflozin for 48 h, as indicated.

### WST-8 assays

The WST-8 proliferation and cytotoxicity assay kit was used to ascertain cell viability in different groups, as previously described. It was performed as reported previously [10].This enabled cell viability to be calculated, according to absorbance.

### Western blot

Western Blot was performed as described [11]. After all of these procedures, the membranes were detected via an ECL system, as shown.

### Flow cytometry

Cell apoptosis was detected by flow cytometry, utilising the Annexin V-PE/7-AAD assay kit. By way of overview, cells were re-suspended into single-cell suspension; 5µL Annexin V-PE and 5µL 7-AAD were added to a 500µL suspension with a total number of cells at 1*10^4^. Cells were then incubated at 37℃ for 15 min. The apoptosis rate was subsequently detected via BD LSRFortessa.

### Phalloidine staining

Cells were cultivated on glass coverslips. After completing the aforementioned process (above), coverslips were taken out and fixed with 4% paraformaldehyde for 15 min. The coverslips were then permeated with 0.3% triton and incubated with a goat serum working solution at room temperature for 30 min. After blocking, coverslips were stained with Phalloidine at a suitable concentration for 1 h. Thereafter, the coverslips were photographed at 400* field of view.

### Cholesterol detection assay

Cholesterol was assessed by utilising a cholesterol detection assay kit (E1015, Polygen company of Beijing, China) and was conducted as per the manufacturer’s instructions.

### Nile red staining

Podocytes were seeded in a 24-well plate at a density of 5 × 10^4^ per well, and placed in a humid incubator for the first night. Cells were treated as described above for another 48 h. Before cells were stained with Nile acid, cells were washed with PBS on three separate occasions. After staining for 5 min, the 24-well place was examined via a fluorescence microscope. Pictures were captured at the randomised field.

### Immunofluorescence

Cells grown on glass coverslips were treated as previous described, in line with the experimental conditions. After the time was up, coverslips were fixed with 4% paraformaldehyde for 20 min, followed by permeability with 0.3% triton for 10 min. After incubating the cells with goat serum working solution to block nonspecific binding for 1 h at 37 °C, the cells were then incubated with a primary antibody of a suitable concentration at 4 °C overnight. Then, the cells were stained with a DyLight594-conjugated secondary antibody for 1 h at room temperature. Following this, the cells were then incubated with 4, 6-diamidino-2-phenylindole (DAPI) for 5 min, in order to stain the nuclei. Randomly-selected fields were subsequently imaged using a fluorescent microscope.

### Transfection of si-KLF-5

Small interference RNA (siRNA) specific for KLF-5 (si-KLF-5) was designed and purchased from Genomeditech company (Shanghai). Cells were cultured in a 6-well plate. Cells were transfected with Opti-MEM medium containing si-KLF-5/NC and Lipofectamine™ 2000 for 6 h when cells reached approximately 60–70% confluency. Then cells were changed with a fresh complete medium for the whole night. The next day cells were then treated with high glucose or high glucose plus dapagliflozin (5, 10µM). Si-NC transfected cells were also grown in the same condition.

### Double luciferase reporter gene assay

For the double luciferase reporter gene assay, the predicted binding sequence was identified and downloaded from the JASPAR database. A recombinant H-ABCG1 promoter plasmid containing the binding sequence was constructed with the help of GenePharma (Shanghai, China). Two recombinant H-ABCG1 promoter plasmids containing the mutant site sequence were also established. Overexpression plasmid for KLF-5 was also generated. Enzyme activity was measured via the Luciferase Reporter Assay System (Promega, Madison, WI) in accordance with the manufacturer’s instructions.

### Animal assay

Four-week-old male Wistar rats were purchased from the Animal Experimental Centre of Shandong University. The experimental protocols for all animal studies were approved by the Animal Ethics Committee of Shandong Provincial Hospital, affiliated with Shandong First Medical University. After accommodations lasting for one week, rats were randomly divided into three groups (8 rats per group). The control group was provided with a normal diet. The diabetic nephropathy (DN) group was arranged with STZ intraperitoneal injections until proteinuria appeared. These were fed with a high glucose/fat diet (feed formulation: 67% ordinary feed + 10% lard + 20% saccharose + 2.5% cholesterol + 0.5% sodium cholate). The Dapagliflozin group was fed via gavage on the basis of DN. All the experiments lasted approximately 12 weeks, after which time the rats were killed. Serum was collected and stored at -80 °C for the detection of biochemical and other indicators. A section of the renal tissue was cryopreserved for quantitative detection, whereas another section of the renal tissue was fixed with formaldehyde for morphological staining.

### Histopathological and immunohistochemical analysis

Extracted tissue samples were cut and fixed in 10% neutral formalin for 48 h. Then, they were each embedded in paraffin. Tissue sections up to 4–6 μm thick were obtained. Following these preliminary steps, deparaffinized sections were stained with hematoxylin and eosin (H&E), PAS, and Masson’s trichrome solution in order to facilitate analysis of histopathological characteristics.

Prior to investigating the immunohistochemistry of the samples, the tissue sections were heated using citrate buffer (pH 6.0), helping to unmask antigens. Samples were pre-treated with a 3% hydrogen peroxide solution in order to block endogenous peroxidase activity. Secondary goat anti-mouse or anti-rabbit antibodies (Dako, Carpinteria, CA, USA) were applied as appropriate to detect primary anti-nephrin (1:500), anti-podocin (1:500), anti-KLF-5(1:500) and anti-ABCA1 (1:500) antibodies. Primary antibodies were incubated within a humidity chamber overnight at 4 °C, after blocking the slides for 30 min with 3% BSA. DAB (Dako) was employed as the HRP substrate for signal detection.

### Statistical analysis

Data herein are presented as the mean ± S.D. unless stated otherwise. One-way ANOVA was utilised in order to determine statistical differences between groups. Dunnett’s Test was also employed, enabling us to perform multiple comparisons between groups. Two-tailed *P* < 0.05 indicates statistical significance. All statistical analyses were performed using SPSS 20.0 software (SPSS Inc. Chicago, Illinois, USA).

## Results

### Dapagliflozin effectively alleviates cell injury induced by high glucose

**Fig. 1:**
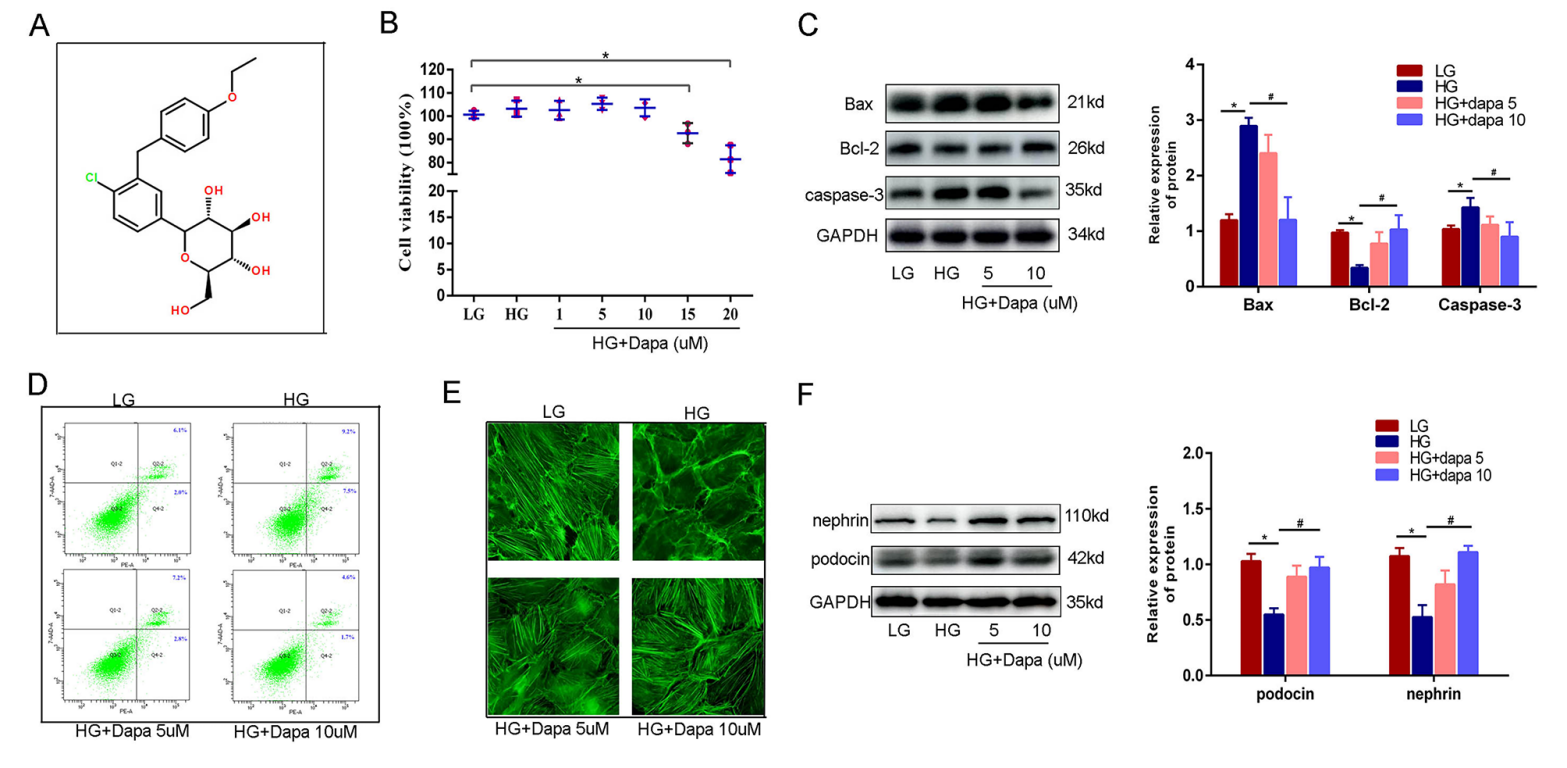
Dapagliflozin could effectively alleviate cell injury induced by high glucose. (**A**) The chemical structure of dapagliflozin. (**B**) WST assay shows that there was no significant difference in cell viability when concentration of dapagliflozin was less than 10µM. (**C**) WB revealed that dapagliflozin could inhibit apoptosis which was induced by high glucose. (**D**) FCM showed dapagliflozin could relieve the apoptosis ratio. (**E**) The cytoskeleton of HPCs stained by FITC-phalloidin showed that the rearrangement induced by HG can be efficiently restored by dapagliflozin. (**F**) WB showed dapagliflozin could relieve cell injury induced by high glucose. (Data presented are the mean ± S.D.; *P < 0.05 vs. LG, # *P* < 0.05 vs. HG, *n* = 3)

Dapagliflozin was the first SGLT2 inhibitor that was approved by FDA to treat diabetes mellitus, the chemical structure of which is illustrated in Fig. 1A. First, a CCK-8 experiment was conducted in HPC to assess the safety of the dapagliflozin dosage. As exhibited in Fig. 1B, concentrations below 10 μm were found to be relatively safe. As the concentration increased, cytotoxicity became concomitantly reinforced. On this basis, 5 and 10 μm were selected for the following experiments. WB and FCM were used to evaluate podocyte apoptosis. As illustrated in Fig. 1C, WB reveals that dapagliflozin has the potential to significantly restore the expression of Bax, Bcl-2 and caspase 3 which were induced by high glucose. In addition, dapagliflozin could also mitigate the apoptosis ratio induced by high glucose with FCM, as illustrated in Fig. 1D. Furthermore, phalloidine staining intuitively revealed that in low glucose contexts, the cytoskeleton was spindle-shaped in the same direction. In contrast, F-actin lined the perimeter of the cell in high glucose contexts. Dapagliflozin pre-treatment prior to the onset of high glucose contexts could thus significantly recover the cytoskeleton in a concentration-dependent manner (Fig. 1E). Last but not the least, dapagliflozin was able to upregulate the expression of nephrin and podocin, which were downregulated in high glucose contexts (as exhibited in Fig. 1F).

### Dapagliflozin can reduce cholesterol accumulation by upregulating the expression of ABCA1, which could promote cholesterol efflux

**Fig. 2:**
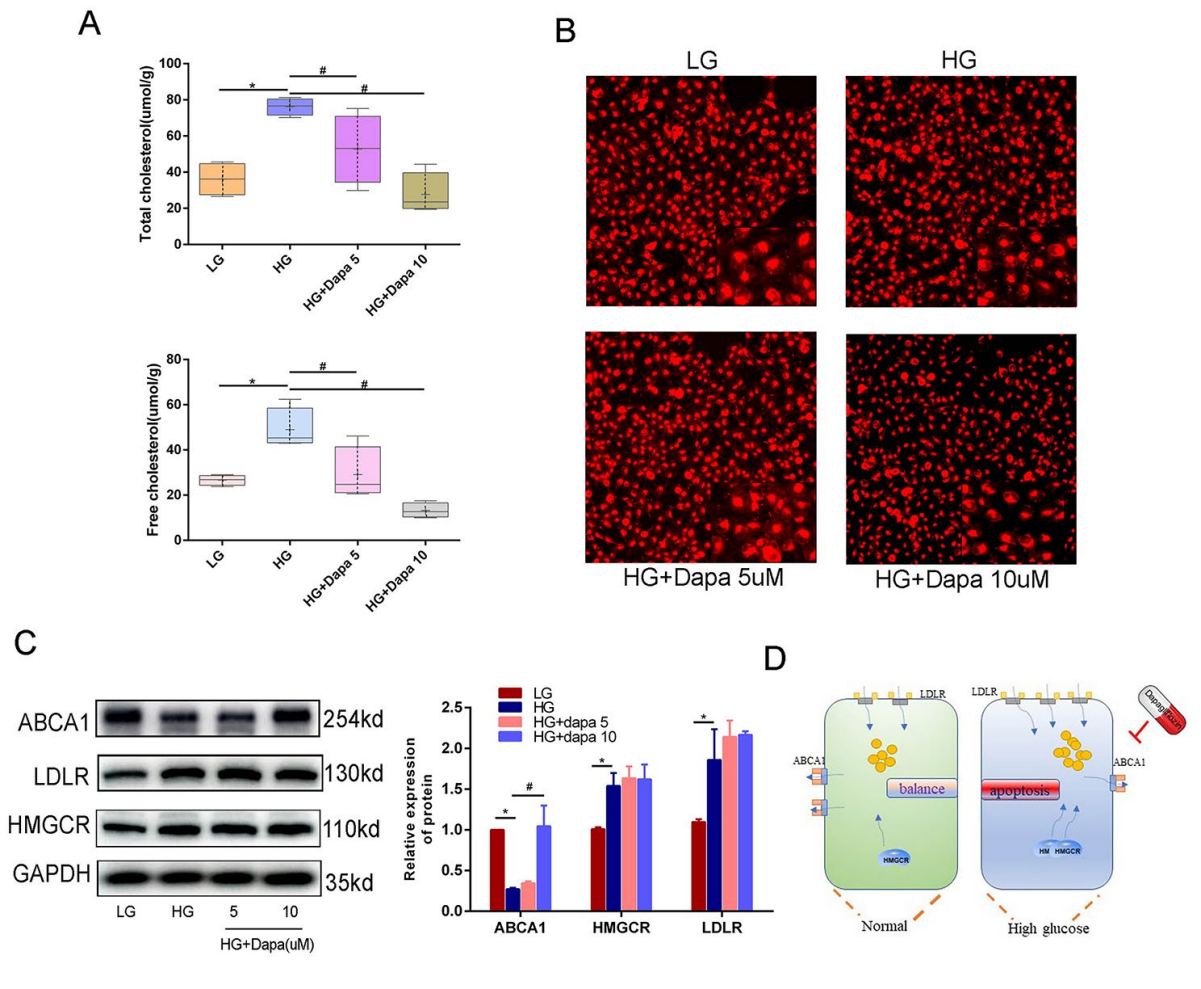
Dapagliflozin could reduce cholesterol accumulation by promoting cholesterol efflux. (**A**) The cholesterol test kit showed dapagliflozin could relieve cholesterol accumulation (both total cholesterol and free cholesterol) which was induced by high glucose. (**B**) Nile red staining showed that high glucose induced lipid accumulation could be relieved by dapagliflozin. (**C**) WB showed that dapagliflozin could restore the expression of ABCA1 but have no effect on LDLR and HMGCR. (**D**) schematic diagram showed that the protective mechanism of dapagliflozin on podocyte lipotoxicity was promoting cholesterol efflux. (Data presented are the mean ± S.D.; *P < 0.05 vs. LG, # *P* < 0.05 vs. HG, *n* = 3)

Next, in order to further evaluate the mechanism underpinning the renoprotective effect of dapagliflozin, lipotoxicity became the focus of our research. As shown in Fig. 2A, dapagliflozin reduced cholesterol accumulation which was induced by high glucose in a concentration-dependent manner. Nile red staining clearly demonstrates that dapagliflozin can mitigate the accumulation of lipids, as illustrated in Fig. 2B. The content of cholesterol was demonstrably controlled by synthesis, influx, and efflux. The key molecules of these processes are HMGCR, LDLR, and ABCA1 (respectively). Hence, we evaluated their expression level via WB. As detailed in Fig. 2C, dapagliflozin showed potential in restoring cholesterol efflux whilst remaining invalid in cholesterol synthesis and influx. Specifically, it could increase the expression of ABCA1, which was decreased in high glucose contexts, while the upregulation of LDLR and HMGCR under high glucose conditions was not alleviated by dapagliflozin. In sum, our team came to the conclusion that dapagliflozin can relieve cholesterol accumulation by promoting efflux via upregulating the expression of ABCA1 (Fig. 2D).

### Dapagliflozin can restore the expression of KLF-5 which was downregulated in high glucose contexts

**Fig. 3:**
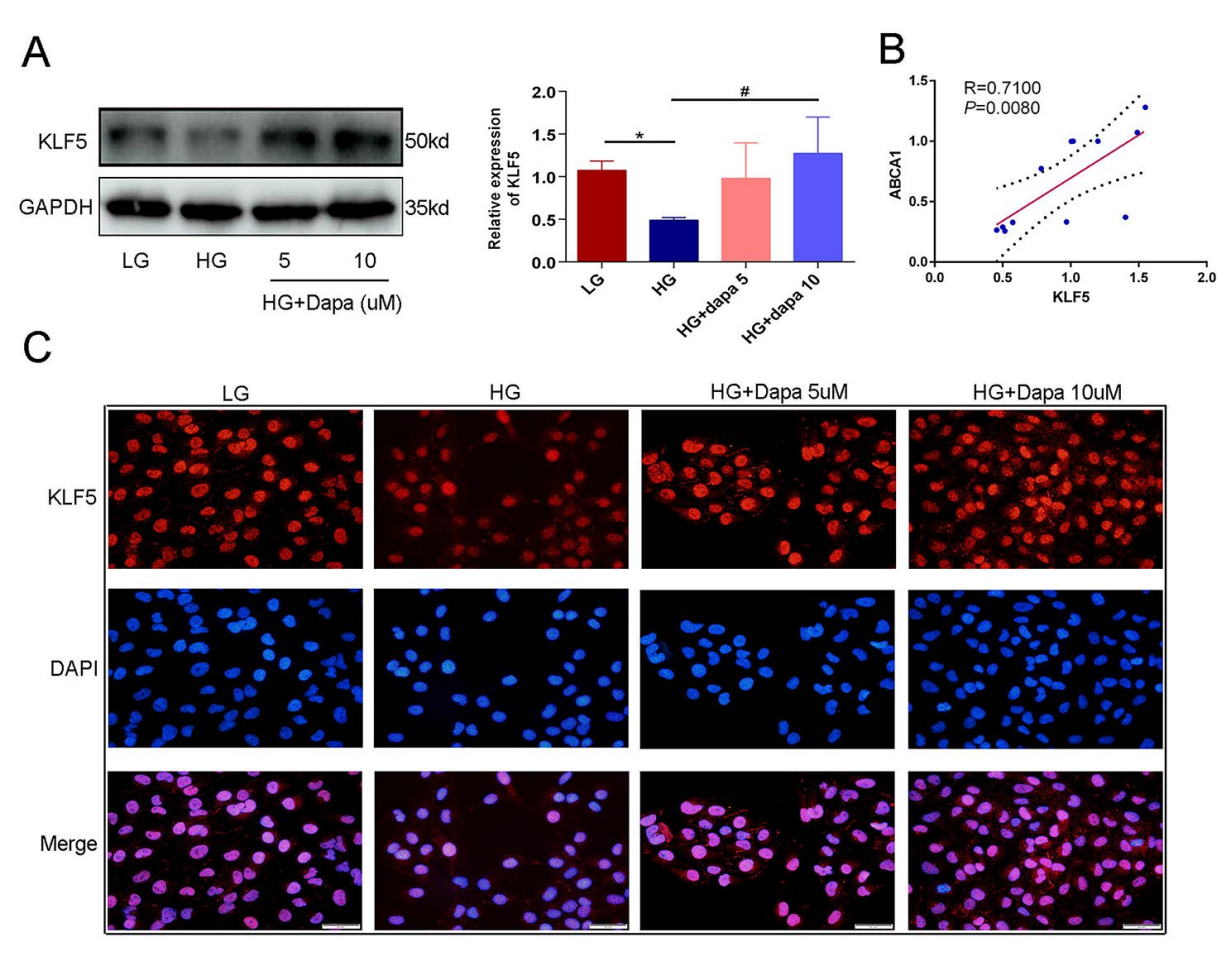
Dapagliflozin could restore the expression of KLF-5 which was downregulated in high glucose. (**A**) Dapagliflozin could restore the expression of KLF-5 which was inhibited in high glucose. (**B**) Pearson correlation analysis showed the expression of KLF-5 and ABCA1 exhibited high correlation. (**C**) IF showed that the downregulation of KLF-5 in high glucose could be upregulated significantly by dapagliflozin treatment. (Data presented are the mean ± S.D.; *P < 0.05 vs. LG, # *P* < 0.05 vs. HG, *n* = 3)

KLF-5 is a vital molecule integral to the metabolic function of lipids. In order to ascertain whether it participates in the protective effect of dapagliflozin, its expression was detected across various aspects. First, WB revealed that dapagliflozin could upregulate the expression of KLF-5, which was previously downregulated in high glucose contexts (Fig. 3A). To evaluate the correlation between ABCA1 and KLF-5, the Pearson correlation coefficient (r) was employed. As presented in Fig. 3B, a high correlation between ABCA1 and KLF-5 is found, with *R* = 0.71 (*P* = 0.008). IF revealed that high glucose contexts could reduce the fluorescence intensity of KLF-5, whereas dapagliflozin could restore its expression(Fig. 3C).

### KLF-5 mediated the protective effect of dapagliflozin on podocyte injury

**Fig. 4:**
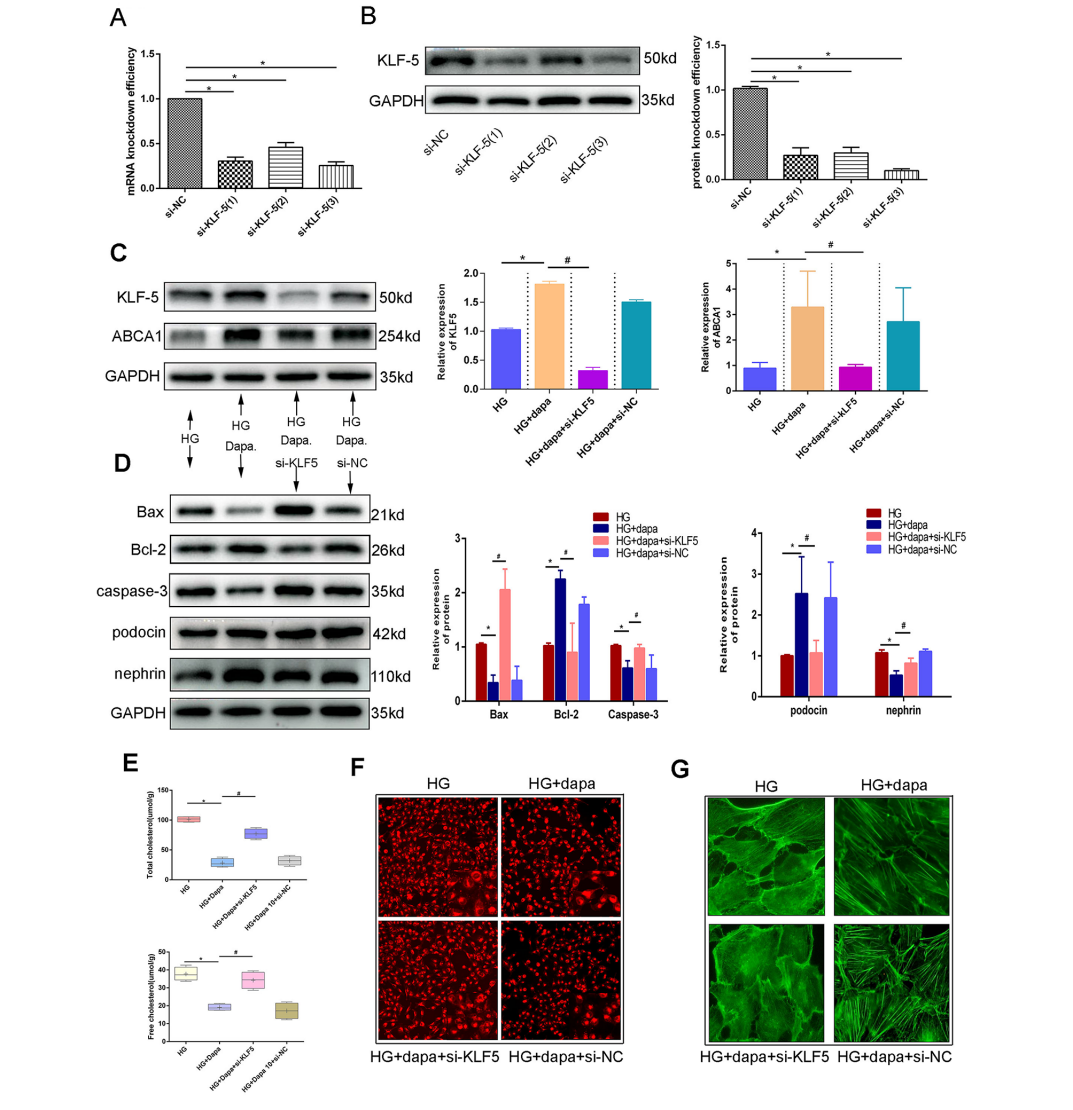
KLF-5 mediated the protective effect of dapagliflozin in podocytes injury. (**A**) RT-PCR was employed to detect the knockdown efficiency of three different si-RNA specific for KLF-5(si-KLF-5). (**B**) WB was also used to detect the knockdown efficiency. (**C**) KLF-5 rescue experiments showed that si-KLF-5 could rescue the expression of ABCA1, which was upregulated in high glucose plus dapagliflozin. (**D**) The rescue experiment showed that si-KLF-5 on the basis of high glucose plus dapagliflozin could accelerate apoptosis and cell injury. (**E**) It is found that si-KLF-5 could restore the amount of cholesterol in podocyte on the basis of high glucose plus dapagliflozin when compared with high glucose plus dapagliflozin group. (**F**) Nile red showed similar results as (**E**). (**G**) Phalloidin staining showed KLF-5 mediated the effect of dapagliflozin on cytoskeleton. (Data presented are the mean ± S.D.; *P < 0.05 vs. HG, # *P* < 0.05 vs. HG + dapa, *n* = 3)

In order to verify the important role of KLF-5 in mediating the effect of dapagliflozin, si-KLF-5 was established. RT-PCR was employed in order facilitate identification of the silencing efficiency. As illustrated in Fig. 4A, both si-KLF-5(1) and si-KLF-5(3) exhibit high levels of silencing efficiency (≥ 70%) relative to si-KLF-5(2). Moreover, WB was also utilised to identify the silencing efficiency at the protein level. As revealed in Fig. 4B, si-KLF-5(3) shows a relatively high silencing efficiency. Concordantly, in the following experiments, si-KLF-5(3) was chosen. Figure 4C shows that the silence of KLF-5 by si-KLF-5 could downregulate the high expression of ABCA1, which was induced by dapagliflozin. In order to further reveal the extent to which KLF-5 could mediate the effect of dapagliflozin on apoptosis, cholesterol accumulation, and cell injury, rescue experiments were subsequently conducted. Figure 4D demonstrates a decreased expression of Bax, whereby caspase 3 induced by dapagliflozin could be rescued by si-KLF-5. Meanwhile, the increased expression of Bcl-2, podocin and nephrin could also be rescued by si-KLF-5 relative to si-NC. In terms of lipotoxicity, si-KLF-5 could rescue the protective effect of dapagliflozin on the content of total cholesterol and free cholesterol (Fig. 4E). Figure 4F clearly illustrates this phenomenon. Furthermore, si-KLF-5 could aggravate cytoskeleton rearrangements, even in the presence of dapagliflozin, as demonstrated in Fig. 4G.

### Dapagliflozin improved apoptosis, injury and cholesterol accumulation in glomerulus of DN

**Fig. 5:**
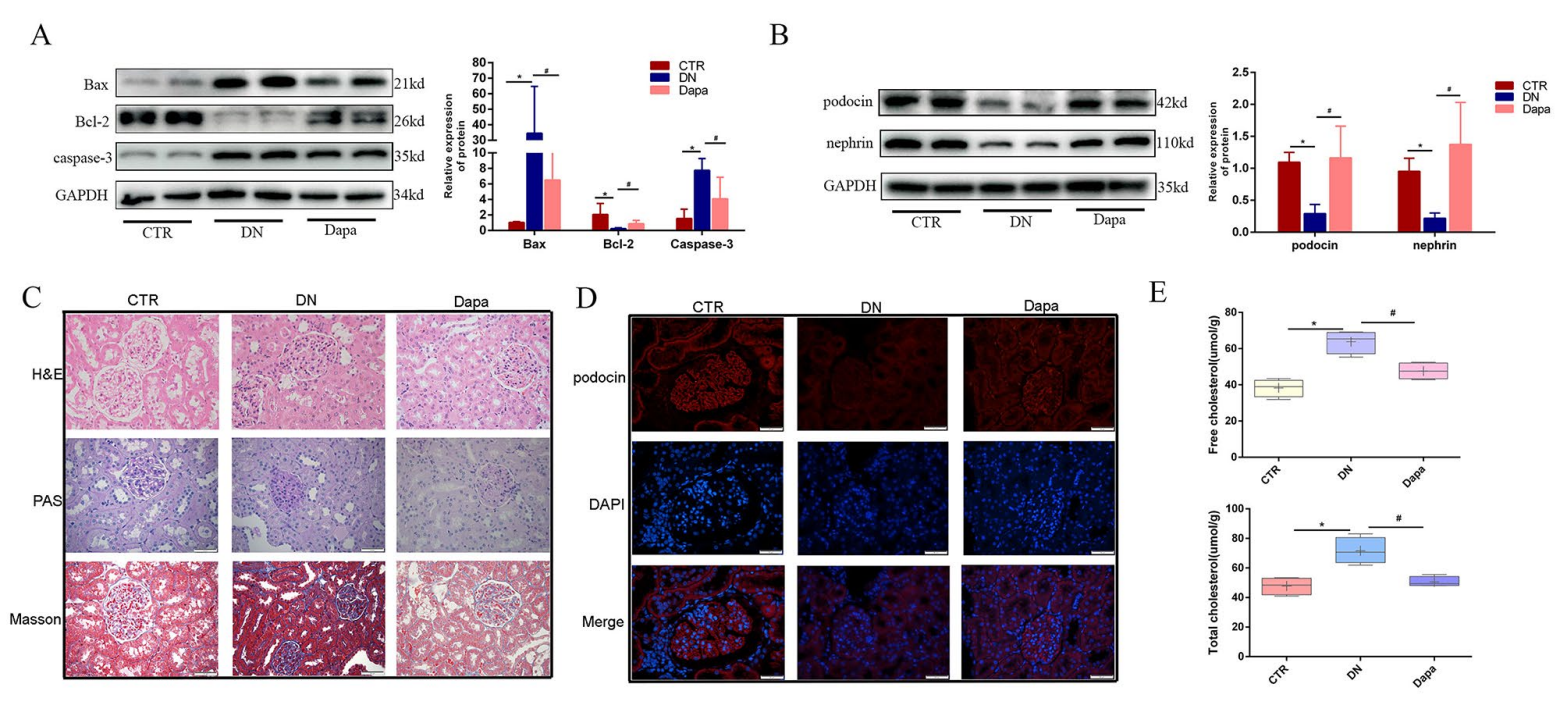
Dapagliflozin improved apoptosis, pathological injuries and cholesterol accumulation in glomerulus of DN. (**A**) WB revealed that dapagliflozin treatment could relieve apoptosis which were induced in DN group. (**B**) Dapagliflozin could restore the expression of podocin and nephrin compared with DN group. (**C**) HE, PAS and MASSON staining showed dapagliflozin could alleviate pathological injuries compared with DN group. (**D**) IF showed dapagliflozin treatment could restore the expression of podocin. (**E**) Cholesterol detected in serum showed that dapagliflozin could significantly reduce the content when compared with DN group. (Data are the mean ± S.D.; *P < 0.05 vs. CTR, # *P* < 0.05 vs. DN, *n* = 3)

We also identified the protective effect of dapagliflozin on apoptosis, injury and cholesterol accumulation of glomerulus in vivo. First, in terms of apoptosis, WB revealed that apoptosis in the DN group was aggravated, whereas dapagliflozin could effectively relieve it. As further shown in Fig. 5A, dapagliflozin could decrease the expression of Bax and caspase 3 which were upregulated in the DN group. Moreover, it could restore the expression of Bcl-2 significantly which was downregulated in the DN group. Second, in terms of glomerular injury, WB revealed that dapagliflozin could increase the expression of podocin and nephrin as shown in Fig. 5B. In addition, in Fig. 5C, morphological staining revealed that in the DN group, the glomerulus manifests significant hypertrophy, mesangial hyperplasia, a loss of normal shape, and fibrosis. While in the dapagliflozin group, pathological morphology is found to be significantly improved and tended towards normal morphology. IF staining further revealed that dapagliflozin could restore the expression of podocin, as illustrated in Fig. 5D. Third, the content of cholesterol was also detected. As shown in Fig. 5E, dapagliflozin shows potential to significantly decrease the expression of both total and free cholesterol, which were both upregulated in the DN group.

### Dapagliflozin upregulated the expression of ABCA1 and KLF-5 in DN

**Fig. 6:**
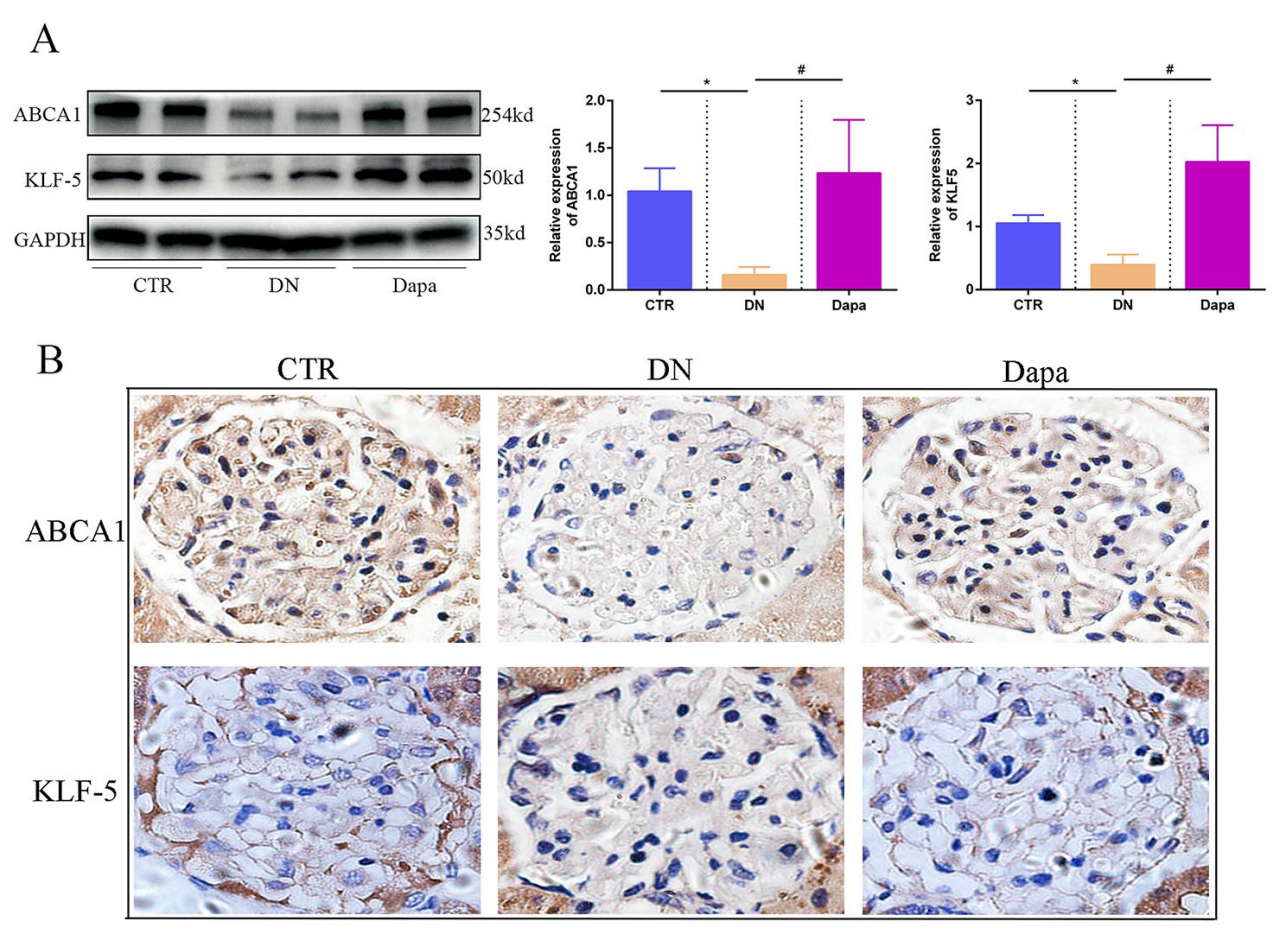
Dapagliflozin upregulated the expression of ABCA1 and KLF-5 in DN. (**A**) WB revealed that dapagliflozin could upregulate the expression of ABCA1 and KLF-5, which were downregulated in the DN group, significantly during in vivo experiments. (**B**) IHC showed that the expression of ABCA1 and KLF-5 were significantly downregulated in the DN group, while dapagliflozin treatment could upregulate it. (Data presented are the mean ± S.D.; *P < 0.05 vs. CTR, # *P* < 0.05 vs. DN, *n* = 3)

We further identified whether dapagliflozin could restore the expression of ABCA1 and KLF-5 in vivo. As per Fig. 6A, WB reveals that dapagliflozin significantly increased the expression of ABCA1 and KLF-5, which decreased in the DN group. We also conducted IHC of ABCA1 and KLF-5, as shown in Fig. 6B. Our findings reveal that the expression of ABCA1 and KLF-5 were significantly downregulated in the DN group; meanwhile, the dapagliflozin treatment could effectively increase their expression, corroborating the results of WB.

### KLF-5 is the transcription factor of ABCA1

**Fig. 7:**
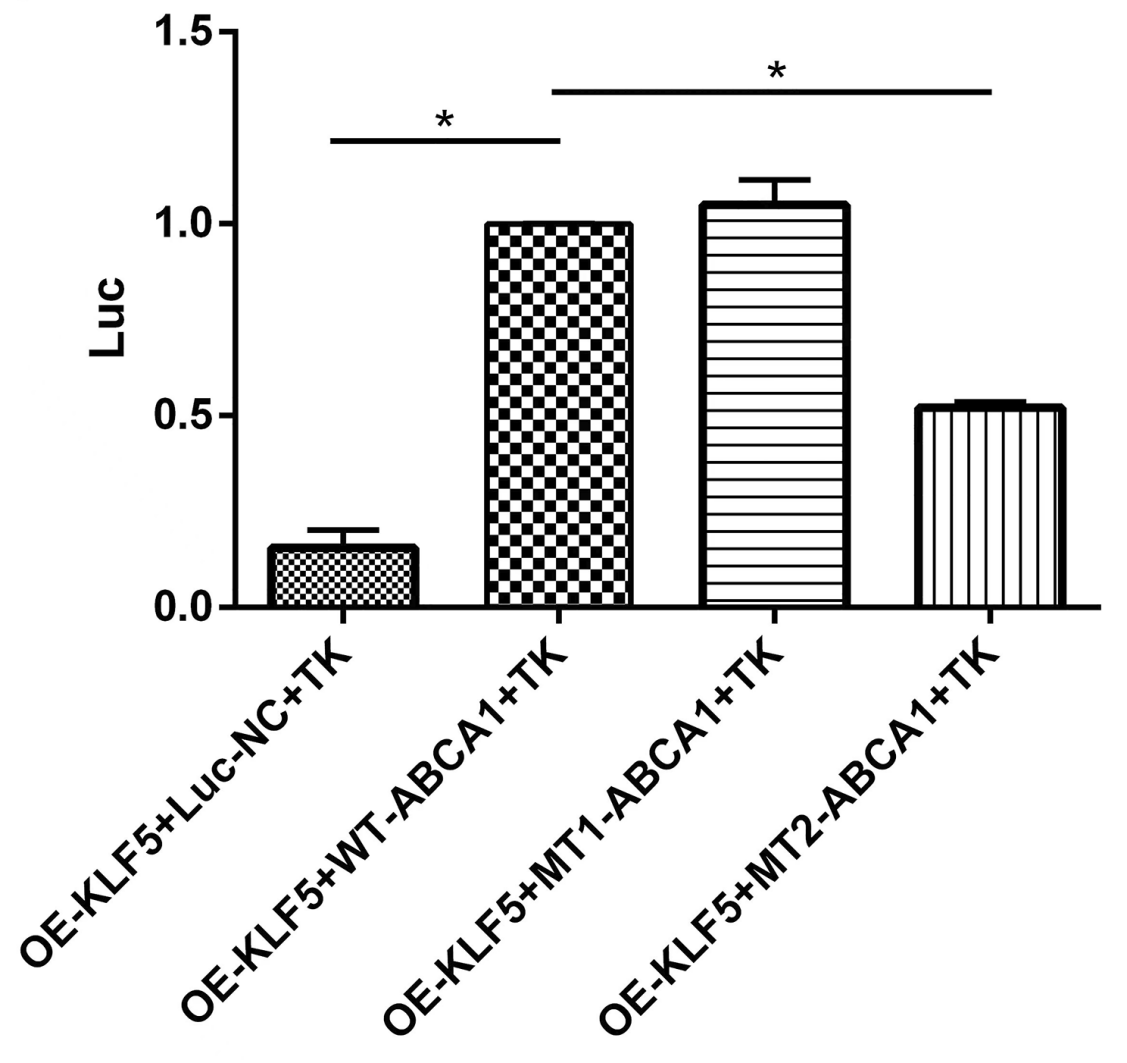
KLF-5 is the transcription factor of ABCA1. The double luciferase reporter gene assay showed that KLF-5 could bound to the promoter region of ABCA1, while mutant 2 ABCA1 is invalid. (Data presented are the mean ± S.D.; *P < 0.05 vs. OE-KLF5 + WT-ABCA1 + TK, *n* = 3)

In order to further verify the specific molecular mechanism of KLF-5 and ABCA1 in podocyte injury of DN, the following experiments were undertaken. First, the NCBI website was used to ascertain the promoter region of ABCA1, and the JASPAR website was consulted in order to predict transcription factors that may be bounded. Among all predicted transcription factors, KLF-5 was lied in and the predicted score was relatively high. Following this, the double luciferase reporter gene assay was utilised to verify the relationship between KLF-5 and ABCA1. As shown in Fig. 7, over-expression of KLF-5 demonstrates a propensity to bind to the wild-type ABCA1 plasmids. Moreover, over-expression of KLF-5 also shows potential to bind to the mutant type I ABCA1 plasmids, but not to mutant type II ABCA1 plasmids. This reveals that KLF-5 could be the transcription factor of ABCA1.

## Discussion

The present study has revealed the protective effect of dapagliflozin on DN in vivo and in high glucose-induced podocytes cultured in vitro. The findings herein demonstrate that dapagliflozin shows great potential in significantly ameliorating the injury of DN and podocytes mediated by relieving cholesterol accumulation via the KLF-5/ABCA1 pathway. Dapagliflozin can inhibit apoptosis, cytoskeleton rearrangement and cholesterol accumulation via KLF-5 in high glucose-induced podocytes cultured in vitro. We have demonstrated that dapagliflozin can also reduce apoptosis, glomerular injury and cholesterol accumulation in DN animal models. Our results thus find dapagliflozin to be a highly promising agent in dealing with DN, and perhaps more pertinently, it even shows potential for reversing changes resulting from injury. Understanding the underlying mechanisms of the renoprotective effects of dapagliflozin shall shed new and much-needed light on the use of SGLT2 inhibitors in the future.

Inhibitors of sodium-glucose cotransporter 2 (SGLT2) are a new type of antidiabetic agents which are independent of pancreatic β-cell function or insulin sensitivity [12]. Dapagliflozin, a highly regarded therapeutic drug targeting proximal tubules, is a selective inhibitor of SGLT2 and significantly slows the progression of kidney disease in type 2 diabetic patients [13]. Initially, it was approved for treating diabetes mellitus by the FDA due to its hypoglycemic effects by inhibiting SGLT2 on proximal tubules and promoting glucose excretion through urine. Later research found it to be effective against renal fibrosis in DN, and even in non-DN [14]. Initially, it was presumed that the diminished glucose levels in circulating blood could be attributed to the kidney protective effect of SGLT2 inhibitors. Yet as research progressed, it became clear that dapagliflozin has a renal protection mechanism independent of hypoglycemia. Over the past few years, significant research efforts have been undertaken in an attempt to clarify the related mechanisms. At the very beginning, the first research into the SGLT2 inhibitor demonstrated that tofogliflozin, reduced albuminuria and glomerular hypertrophy in db/db mice mediated by inhibiting inflammation and oxidative stress; nonetheless, the related mechanism was not reported [15]. Later blowout researches emerged in this field. More recently, professor Vadim V Klimontov demonstrated that the anti-albuminuric effect of EMPA could be attributed to mitigation of podocyte injury and enhancement of nephrin expression so as to attenuate podocytopathy in experimental diabetic kidney disease [16]. In this present research, we have found that dapagliflozin demonstrates kidney protective effects on podocytes by relieving cholesterol accumulation via upregulation of ABCA1 which is a key transporter that moves cholesterol out. To the best of our knowledge, this is the first research which directly engages in dapagliflozin on cholesterol accumulation in human podocytes.

Lipotoxicity is presently a hot topic in the research field of DN [17], with numerous studies demonstrating that it plays an integral role in the progression of the disease. The so-called lipotoxicity refers to the abnormal deposition of lipid components in non-adipose tissue, such as the heart, muscles and kidneys. In circumstances of diabetes mellitus, excessive high glucose, which is not converted into glycogen, would inevitably alter the energy-sensing mechanism. As we suspected, and as research has revealed, excessive glucose would consequently be stored as metabolic energy in the form of lipid content. Concomitantly, a series of pathological effects would occur, such as ROS generation, mitochondria injury and oxidative stress. In a narrow sense, lipid primarily contains fatty acid and cholesterol. In the physiology state, fatty acids and cholesterol are integral contents of cells and bodies. Once excessive lipid accumulation occurs, oxidative stress, inflammation, apoptosis, and similar morbidities would arise. Compare to fatty acids, cholesterol is more pivotal in podocytes in order to maintain normal conditions, given the podocytes’ special structure. The slit diaphragm formed by the foot process possesses many lipid-raft-like structures. These special structures assemble multiprotein complexes with essential podocyte proteins, such as nephrin (transmembrane proteins), podocin (integral membrane proteins), a-actinin-4 (structural proteins), CD2AP (signalling adaptors), and TRPC6 (ion channels) [17]). For the majority of cases, their interaction is contingent upon cholesterol to assure proper localization and function of slit diaphragm proteins. When lipid/cholesterol imbalance happens, it would perturb cellular functions, leading to other pathomechanistic sequels.

The balance of cholesterol is maintained by inner synthesis, influx and efflux. In this present research, we have discovered that the high content of cholesterol in podocytes induced by high glucose was significantly reduced by the addition of dapagliflozin and the decreased ABCA1 expression in podocytes induced by high glucose and in diabetic mice was reversed by dapagliflozin. ABCA1 is a transmembrane protein, which transports intracellular cholesterol and phospholipids to apolipoproteins on the cell surface [18]. ABCA1 deficiency therefore leads to accumulation of cholesterol as well as phospholipids. Consistent with our research, multiple studies have shown that ABCA1 plays a vital role in podocyte injuries by regulating the efflux of cholesterol [14, 19, 20]. In addition, it has been demonstrated that ABCA1 deficiency contributes to cardiolipin accumulation and mitochondrial dysfunction, leading to podocyte injury and to the progression of DKD [19]. This may also be a potential mechanism through which dapagliflozinsafeguards podocytes via ABCA1. the protective effect of dapagliflozin on ABCA1 expression in podocytes exposed to HG was effectively neutralised by KLF-5 siRNA. These novel findings suggest that dapagliflozin alleviates podocyte injury through inhibition of cholesterol accumulation via upregulation of KLF-5/ABCA1 in DN.

It is widely believed that HDL particles specifically interact with ABCA1 and ABCG1 to promote cholesterol efflux and low HDL levels would limit cholesterol efflux [18]. Previous studies have shown that the administration of dapagliflozin could increase serum HDL levels in patients and in experimental animals [21, 22]. In cultured podocytes, we revealed that dapagliflozin could increase podocyte ABCA1 expression and promote cholesterol efflux in a high-glucose environment without the effect of HDL levels. The increased serum HDL level could also contribute to podocyte cholesterol efflux via ABCA1 in vivo after using dapagliflozin.

Krüppel‑like factors (KLFs) are a group of transcriptional regulatory factors which have an integral role in cell proliferation, migration, apoptosis, and tissue remodelling [23, 24]. KLF-5, a member of the KLF family, can be regulated in multiple ways, such as via phosphorylation, acetylation and ubiquitination [25, 26]. Prior research has demonstrated that KLF5 is closely associated with kidney diseases, such as hypertensive nephropathy and diabetic retinopathy [27, 28]. However, the impacts of KLF5 on DN are not yet fully understood. In this present study, we have effectively revealed that the expression of KLF-5 is downregulated in HG circumstances, both in vivo and in vitro, whereby dapagliflozin could restore its expression level. With regards to podocytes, KLF-5 is a transcription factor targeting ABCA1, which has been found to relieve lipid accumulation by accelerating cholesterol efflux. Similarly, prior research has proven that in PAN-induced podocytes injury contexts, the expression of KLF-5 is also downregulated and overexpression of KLF5 significantly attenuates this effect via upregulation of cyclin D1 and cmyc expression levels [29]. A further study demonstrated that the downregulation of KLF5 is associated with G1-phase cell cycle arrest [30]. Despite this empirical basis, there are some conflicting results regarding the role of KLF-5 in kidney disease. Professor Zhang has demonstrated that LncRNA MALAT1 could relieve the podocyte injury in DN by upregulating let-7f and inhibiting KLF-5 [31]. In their experiment model, they determined that the expression of KLF-5 was upregulated, whereby inhibition of KLF-5 could relieve cell injury. Their experiment utilised db/db mice and murine podocytes, whereas the distinct models we used in this present research in vivo and in vitro might partly account for the difference in results. The role of KLF-5 in DN thus needs to be further verified, and offers a fruitful avenue of future research.

Our study also presents some limitations. Our in vivo experiments were performed on whole kidney tissues. We did not extract podocytes alone to investigate whether the effect of dapagliflozin on podocytes was consistent with the in vitro experiments. The effect of dapagliflozin on epithelial and mesangial cells also requires further investigation.

In conclusion, we herein reveal that dapagliflozin has the potential to inhibit apoptosis and cell injury in podocytes, induced by high glucose. It can moreover relieve cholesterol accumulation by accelerating cholesterol efflux. In terms of mechanism, we identify that dapagliflozin can restore the expression of KLF-5, which is the upregulating transcription factor of ABCA1.

## Data Availability

All data generated during this study are included in this published article.

## Abbreviations

DN: Diabetic Nephropathy
ESRD: End Stage Renal Disease
SGLT2i: Sodium-Glucose Cotransporter 2 Inhibitor
LG: Low Glucose
HG: High Glucose
Dapa: Dapagliflozin
CTR: Control
ABCA1: ATP binding cassette transporter A1
KLF-5: Krüppel‑like Factors 5
GFB: Glomerular Filtration Barrier
LDLR: Low Density Lipoprotein Receptor
HMGCR 3: Hydroxy 3 Methylglutaryl Coa Reductase
DAPI: 4, 6-Diamidino-2-Phenylindole
STZ: Streptozocin
ANOVA: One-Way Analysis Of Variance
FDA: Food and Drug Administration
WB: Western Blot
Rt-PCR: Real Time Polymerase Chain Reaction
EMPA: Empagliflozin
